# On the Optimal Identification of Tag Sets in Time-Constrained RFID Configurations

**DOI:** 10.3390/s110302946

**Published:** 2011-03-04

**Authors:** Javier Vales-Alonso, María Victoria Bueno-Delgado, Esteban Egea-López, Juan José Alcaraz, Juan Manuel Pérez-Mañogil

**Affiliations:** Department of Information Technologies and Communications, Technical University of Cartagena, Plaza del Hospital 1, 30202 Cartagena, Spain; E-Mails: mvictoria.bueno@upct.es (M.V.B.D.); esteban.egea@upct.es (E.E.L.); juan.alcaraz@upct.es (J.J.A.); juan.manuel.perez.manogil@gmail.com (J.M.P.M.)

**Keywords:** RFID, anti-collision protocols, time-constrained configuration, identification time, tag loss probability

## Abstract

In Radio Frequency Identification facilities the identification delay of a set of tags is mainly caused by the random access nature of the reading protocol, yielding a random identification time of the set of tags. In this paper, the cumulative distribution function of the identification time is evaluated using a discrete time Markov chain for single-set time-constrained passive RFID systems, namely those ones where a single group of tags is assumed to be in the reading area and only for a bounded time (sojourn time) before leaving. In these scenarios some tags in a set may leave the reader coverage area unidentified. The probability of this event is obtained from the cumulative distribution function of the identification time as a function of the sojourn time. This result provides a suitable criterion to minimize the probability of losing tags. Besides, an identification strategy based on splitting the set of tags in smaller subsets is also considered. Results demonstrate that there are optimal splitting configurations that reduce the overall identification time while keeping the same probability of losing tags.

## 1. Introduction

Radio Frequency IDentification (RFID) enables the identification of nearby objects or people by means of Radio-Frequency (RF) signals. The communication takes place between small and inexpensive devices called *tags* which are attached to the items to be tracked, and *readers* which collect and manage information about these items. This process is performed in the coverage area of the reader, or checking area. RFID is increasingly being used to identify and track objects in supply chains and manufacturing process [1]. These scenarios consider a large number of tags attached to items which pass through checking areas, usually carried in sets by conveyor belts, pallets, lorries, *etc*. According to the power supply of the tags, RFID systems are classified as active or passive. The former are used in applications which typically require to sense the environment (e.g., Wireless Sensor Networks). The latter are the most extended in logistic facilities due to their low cost. In passive systems, RFID readers continuously emit electromagnetic waves creating *reading areas*. Passing tags are thereby energized allowing them to send their identifiers back to the reader.

Since the communication between tags and reader use a shared wireless channel, when multiple tags reply simultaneously to a reader, a collision occurs. Therefore, to avoid it, an anti-collision protocol is necessary, and readers implement them. Framed-slotted Aloha (FSA) is one of the most widely used anti-collision protocol by passive RFID systems [1]. As in Slotted-Aloha, time is divided into periods called slots, but all slots are confined to a super-structure called “frame”. The reader starts an interrogation frame by sending a *Query* packet informing the tags about the frame length *K* (the number of slots that make a frame). At every frame, each unidentified tag randomly selects a slot among the *K* slots to send its identifiers to the reader. When more than a single tag select the same slot in the frame, a collision occurs, and the reader is not able to recover the identifier of the tags involved in the collision. Variations of FSA are used for instance by ISO/IEC 18000-6C, ISO/IEC 18000-7 [2] and EPCGlobal Class 1 Gen 2 (EPC-C1G2) [3], used by most commercial passive and active RFID systems.

Three different classes of scenarios of practical interest arise in RFID facilities, according to the way the tags behave in the reading areas:

1. **Static scenario**: a group of tags enters the reading area and remains there until all of them have been successfully identified. Other tags do not enter during that time. As an example, let us think about a conveyor belt controlled by a reader: as long as the reader detects collisions, the conveyor is stopped. Once the reader does not detect tags contending, the conveyor belt runs again, allowing new tags to enter. The goal in the static scenario is usually to minimize the average identification time. A thorough evaluation of this case can be found in our previous paper [4].
2. **Flow scenario**: tags are continuously entering and leaving the checking area, according to some scenario dynamics that defines the arrival process. For example, a conveyor belt continuously running with tags randomly scattered on it. In this case, some tags may leave the reader coverage area unidentified. Thus minimizing the average identification time (as in the prior case) should not be optimization goal—instead it is critical to minimize the probability of losing tags. This case has been evaluated in [5] and [6].
3. **Mixed scenario**: in this case a group of tags enter the checking area and stay there only for a certain time (sojourn time). No more groups enter the checking area until the previous one has left. For example, consider a moving truck with tags grouped in boxes, which are uniformly spaced. Like in the flow scenario some tags may leave the reader coverage area unidentified. This is the case addressed in this paper. Our goal is to compute the minimum suitable sojourn time that guarantee that the percentage of groups correctly identified is above some level. In this paper, this level is termed Identification Confidence Level (ICL).

To conduct the study of mixed scenarios a *Discrete Time Markov Chain* (DTMC) is used to obtain the cumulative distribution function of the identification time of a group of tags of size *N*. From it the ICL is computed as a function of the sojourn time. Moreover, based on the previous results, a splitting strategy of the tag set is considered. That is, given a number of tags *N* entering the checking area we seek to find which is the best size of subsets that minimizes the global duration of the identification process guaranteeing the ICL level.

Overall, the results provided in this paper are useful to help manufacturers and system operators to improve the RFID system performance in mixed scenarios. Designers must rely on physical parameters to control the performance of the system, such as, conveyor belt speed, coverage range, *etc*. The usefulness of the results of this paper is twofold. First, given an ICL bound and a packet tag size *N* the minimum sojourn time is computed in Section 3 and from it the physical parameters of the facility (e.g., conveyor belt speed) can be determined. Second, if tags repackaging is possible the optimal packet size is derived from the results exposed in Section 5. Let us remark that the results derived are valid for any FSA protocol with fixed frame size. This includes most active and passive RFID standards used in logistic currently.

The rest of the paper is organized as follows: a review of the related work is provided in Section 2. The analysis of mixed scenarios is addressed in Sections 3 and 4. Section 5 discusses the feasibility of a splitting strategy in the tag set. Finally, Section 6 points out the main conclusions of the work.

## 2. Related Work

A large number of studies has been conducted in the last years with the aim of evaluating the performance of passive RFID systems. Most of them focused on suggesting new protocols and algorithms to improve the performance of RFID installations. The proposals cover a wide range of topics, with an emphasis in security, e.g., [7–9] and anti-collision protocols, e.g., [10–13]. However, the vast majority of these proposals cannot be implemented in off-the-self readers, due to their high computational cost or due to incompatibilities with the current standards [14]. Hence, additional effort must be also devoted to study configuration and deployment techniques to achieve the best performance of RFID systems with the currently available commercial readers. In [15–18] the authors performed several empirical studies to determine the factors that degrade performance and reliability of UHF RFID systems under EPC-C1G2 standard in a static scenario. In [15] the authors compute the read range by means of simulations, while in [16] the authors show how the bit error rate degrades the EPC-C1G2 performance. In [17], the authors experimentally validate the work in [15] and [16], and explore the extent to which reader configuration options, focused on the physical and medium access control layer can mitigate these factors. From the study, authors suggest that tuning physical layer operational parameters may increase the read rate up to a 33%. In [18] it is shown how the performance in EPC-C1G2 varies widely for different readers.

Some works focus on analyzing the identification process of passive RFID systems. A relevant study is addressed by Vogt in [19], where the author characterizes the identification process of ISO-18000-6C standard [2] as a Markov chain, assuming a static scenario. The author found that the results matched an experimental evaluation using the old I-Code RFID system [20]. However, in [19] the author assumes that those tags already identified in previous frames keep on competing. This is not the case currently, since most FSA derived protocols, including EPC-C1G2, force identified tags to withdraw from the identification process.

In [4] we study the identification performance in static scenarios, but also considering the dynamic frame-length procedure of EPC-C1G2, which is not widely implemented in the commercial readers.

There are only a few works that address the mathematical analysis of the identification process in flow scenarios. In [21] and [6] we analyze the identification performance of RFID systems in scenarios characterized by an incoming flow of tags entering the coverage area of a reader, moving at constant speed (e.g., modeling a conveyor belt) and, considering that new tags can enter the workspace although other tags are still being identified. In [6] we provide a model based on dynamical systems for a general scenario where tags enter the checking area according to some arbitrary random arrival process and move with constant speed. With this model, the average tag loss ratio (tags lost per tags entering) can be computed. Moreover, assuming a fixed frame length identification procedure this model provides the optimal frame length for a given set of configuration parameters (speed, tag density and so on). Let us remark that these results can not be used in mixed scenarios, where new groups of tags do not enter in the reading area during the sojourn time.

A work focused on mixed scenarios has been published [22], where a probabilistic model is provided for a conveyor belt carrying tags grouped in boxes. However, the main difference between [22] and this work is that, in the former the authors consider a dynamic frame length operation and provide a dynamic programming algorithm to optimally adjust the frame length based on their model. But, as stated previously, most commercial readers do not provide the dynamic frame capability and use FSA with a constant frame length. Therefore, the performance has to be improved by adjusting other system’s parameters. In this paper we address this case.

## 3. Identification Process in Mixed Scenarios

The identification performance of FSA RFID systems in static scenarios was addressed by the authors in [4]. In this work we begin reviewing the Markov analysis of that scenario since it is the basis of the controlled-arrival scenario.

Static scenarios are characterized by a block of tags (modeling a physical container like a pallet, a box, *etc.*) that enter the checking area and remain there until all of them are successfully identified. Two related performance metrics are commonly considered:

- The average identification time, defined as the mean number of time units (slots, frames, seconds, *etc.*) until all tags are identified.
- The system throughput or efficiency, defined as the inverse of the mean identification time, *i.e.*, the ratio of identified tags per time unit.

For mixed scenarios where the tags only remain in coverage for a certain sojourn time the goal must be different. In this case, the probability that some tag lost in a group must be minimized. As stated in the introduction, configuration must be established to ensure with a given Identification Confidence Level (ICL) that all the tags in a set have been identified before leaving the reading area. Given an ICL level the minimum required sojourn time can be derived from the cumulative distribution function of the reading time, which is computed in the next sections.

### 3.1. Markovian Analysis

**Figure 1.**
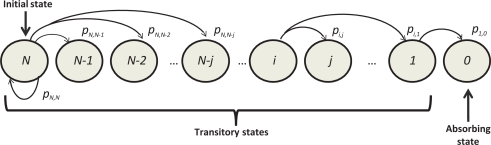
Partial Markov Chain.

The state of an identification process in a static and a mixed scenario is determined by the number of remaining unidentified tags. Thus, the identification process can be modeled as a homogeneous DTMC, *X_s_*, where each state of the chain represents the number of unidentified tags, being *s* the frame number. Thus, the state space of the Markov process is {*N*, *N* − 1, . . . , 0}, being *N* the number of tags to identify. Figure 1 shows a partial DTMC state diagram from the initial state, *X*_0_ = *N*. The transitions between states is governed by the probability of identifying a certain quantity of tags *t* in a given frame or, in other words, the probability of going from state *i* to an state with (*i* − *t*) tags still unidentified. Let us remark again that we are considering the number of tags identified in a frame so a maximum of *t* = *K* tags can be identified in a frame.

The transition matrix *P* (as usual *p_ij_* denotes the probability of going from state *i* to state *j*) depends on the anti-collision protocol used and its parameters. To compute the transition matrix *P* in FSA, let us denote *K* as the number of slots per frame (frame length), and let us define the random variable *μ_t_*, which indicates the number of slots being filled with exactly *t* tags in a reading frame.

When *t* = 1, *μ*_1_ provides the number of slots with a successful identification for a given frame length and contending tags. Its probability mass function is given by (see [23]):

(1) $$P_{K,N}\left(\mu_{1}=m\right)=\frac{K!N!}{m!K^{N}}\sum_{z=0}^{N-m}\frac{{\left(-1\right)}^{z}{\left(K-m-z\right)}^{N-m-z}}{\left(N-m-z\right)!z!\left(K-m-z\right)!}$$

That is, *P_K,N_* (*μ*_1_ = *m*) provides the probability that *m* slots have a single reply with a frame length of *K* slots and *N* tags contending. Recall from the introduction that *K* denotes the number of slots per frame. Thus, taking into account that tags identified in a frame will not contend in the following ones, the transition matrix *P* [24] is:

(2) $$p_{i,j}=\{\begin{array}{ll} P_{K,i}\left(\mu_{1}=i-j\right) & ,i-K\leq j\leq i\\ 0 & ,\text{otherwise} \end{array}$$

for *i* = 0, . . . , *N*.

The Markov chain clearly has a single absorbing state, *X_s_* = 0. That is, all tags are identified after an arbitrary long time period. The mean number of steps until the absorbing state is the mean number of identification frames (*s̄*). It can be computed by means of the fundamental matrix, *D*, of the absorbing chain [25]. *D* is obtained from the canonical form [26] of the transition probability matrix *P*. In our case, since there is only one absorbing state *P* has the form:

(3) $$P=\left(\begin{array}{cc} F & Q\\ 0 & 1 \end{array}\right)$$

where *F* denotes the sub-matrix of *P* with the all the transient states (note that the size of *F* is *N* × *N*). Thus, the fundamental matrix *D* is:

(4) $$D={\left(I_{N}-F\right)}^{-1}$$

being *I_N_* the identity matrix of size (*N* × *N*).

From *D* the average number of identification frames *s̄* can be calculated as follows:

(5) $$\bar{s}=\sum_{y=1}^{N}D_{1,y}$$

That is, the sum of the elements of the first row in *D*.

Besides, the corresponding mean number of slots is *L̄* = *s̄ · K* since the frame length is constant.

### 3.2. Computation of the Minimum Sojourn Time for a Given ICL

**Figure 2.**
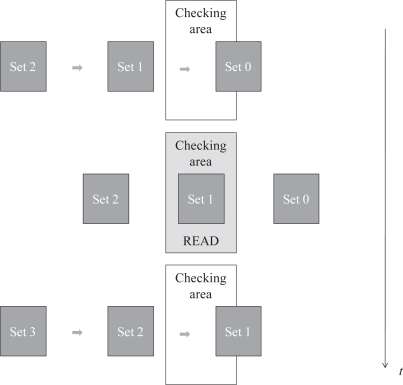
Typical identification procedure in semi-static scenario. A set of tags enter the coverage area and stay for a sojourn time (during which tags are being identified) before a new set enters.

As stated in the introduction, when tags enter the reading area in mixed scenarios new groups of tags do not enter until the previous one has left it. Therefore, the bulk of tags remains in the reading area only for a bounded sojourn time and some tags may be still unidentified after leaving. Figure 2 depicts this operation mode. Hence, for these type of scenarios the goal is *to minimize the number of times this event of losing tags can occur*. Let us remark that the identification time is an unbounded random variable for FSA systems (*i.e.*, the identification time can be arbitrarily high with probability greater than zero). That is, it is not possible to establish a sojourn time which guarantees in the 100% of cases the identification of all the tags. Instead, a minimum sojourn time can be selected to guarantee that in all the cases the probability of identifying all the tags is higher than a given probability threshold, termed as Identification Confidence Level (ICL). From a practical point of view, the design process would be as follows: first, it starts with a quality requirement in the form of a given ICL parameter, then the minimum sojourn time needed to achieve the ICL is computed, and finally, the tunable system parameters (speed, tag population or others) are set to appropriate values to get the desired sojourn time. In the following sections we provide a method to compute the sojourn time for an ICL level.

The analysis and computation of the minimum sojourn time needed to achieve an ICL level uses the DTMC built in the previous section. The Markovian analysis to compute the transition probabilities is the same as in the static scenario. Hence, we start the analysis from the transition probability matrix *P* given in Equation (2). Let us denote $\pi^{\left(s\right)}=\left[\pi_{0}^{\left(s\right)},\ldots ,\pi_{N}^{\left(s\right)}\right]$ the state probability vector of our DMTC at frame *s*, that is, a row vector where the *i*-th element $\pi_{i}^{\left(s\right)}$ represents the probability that *i* tags have been identified in *s* frames. From another point of view, $\pi_{i}^{\left(s\right)}$ is the probability of being at a particular system state *N* − *i* in the frame *s*. Besides, let *π*^(0)^ = [1 0, . . . , 0] be the initial distribution (since none of the *N* tags is identified before identification process starts). Then:

(6) $$\pi^{\left(s\right)}=\pi^{\left(0\right)}\cdot P^{s}$$

That is, the state probability vector at frame *s* is given by the product of the initial distribution and the *s* power of the transition matrix *P*. Let *V_N_* denote the random variable that indicates the number of frames required to identify *N* tags. Its *cumulative distribution function* (CDF) can be computed from Equation (6) as:

(7) $$P\left[V_{N}\leq s\right]=\pi_{N}^{\left(s\right)}$$

From the CDF of *V_N_*, the minimum number of frames *s* required to achieve a given ICL level can be computed. The time elapsed in these frames is the minimum sojourn time needed.

**Figure 3.**
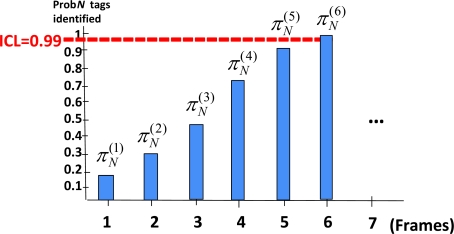
Minimum sojourn time for a given ICL.

Figure 3 illustrates this method for ICL set to 0.99. The *s*-th column represents the probability of having identified the whole set of *N* tags at end of frame *s*, that is $\pi_{N}^{\left(s\right)}$. In this example, after six frames, the probability of identifying all tags is higher than the ICL selected. That is, each group of tags should remain in coverage for at least 6 identification frames in order to guarantee that, in the 99% of the cases, all tags are successfully identified. Let us remark that this is not the same as guaranteeing that 99% of tags are successfully identified in each group, being the former condition stronger that the latter one.

## 4. Reading Process Evaluation

In this section the reading process is evaluated for mixed scenarios. The goal is to point out the differences in the reading performance if the ICL criterion is used to set the sojourn time, rather than using the simpler approximation of setting the average identification time as the sojourn time.

**Figure 4.**
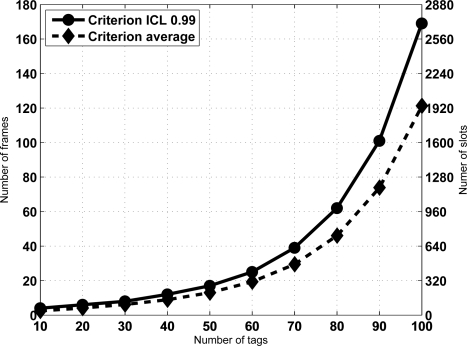
Identification time (frames and slots) needed for different tag populations and *K* = 16. Criterion *ICL* = 0 99 shows the minimum number of frames computed from Equation (7), whereas Criterion average shows the number of frames needed when computed using Equation (5). The number of slots is given by the number of frames times the frame length.

From Equation (7) the sojourn time is computed for an ICL level set to 0.99 assuming a frame length *K* = 16, and for a range *N* from 10 to 100 tags. Figure 4 shows the results. Let us remark that the results are exactly computed from Equation (7), no simulation has been done. Note that sojourn time is expressed as frames, and since frame length is fixed, the conversion to slots is provided as well. Besides, Figure 4 also shows the average number of frames (slots) required for identification, computed from Equation (5). Let us remark that this second curve corresponds to the criterion of selecting the average identification time as the sojourn time. Therefore, it has been referred to as “Criterion average” in the figure. Figure 4 also shows that achieving an ICL level of 0.99 becomes harder as the number of tags increases. In fact, it is the expected result when the frame length *K* is not high enough to cope with them. In that case, the frame length must be increased properly. To illustrate it, Table 1 provides the number of frames needed to achieve an ICL level of 0.99 when *K* = 16, 32 and 64 slots and slots are *N* = 50, 100, 200 and 300 tags. As can be seen, by increasing the frame length according to the expected number of tags it is possible to achieve the given ICL in a reasonable amount of time.

Note from Figure 4 that the derivative of the sojourn time seems to increase with *N*. This has an important implication. Namely, that *the reading efficiency decreases for large sets*. This effect can also be seen in Table 1. In fact, as a result of this observation we propose a identification strategy that improves the reading efficiency by splitting the set of tags in subset of lower size. Section 5 deals with this property and its consequences.

**Figure 5.**
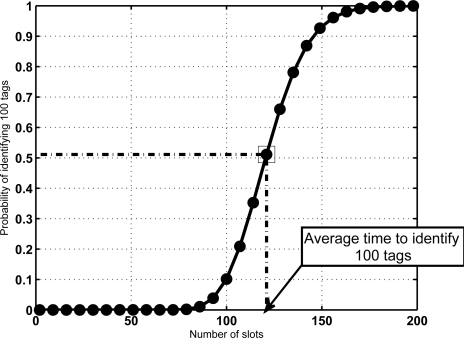
Cumulative Distribution Function for *N* = 100 and *K* = 16. Figure also shows the average number of slots needed to identify 100 tags from Equation (5), which only provides barely a 50% of probability of having identified all the tags.

**Figure 6.**
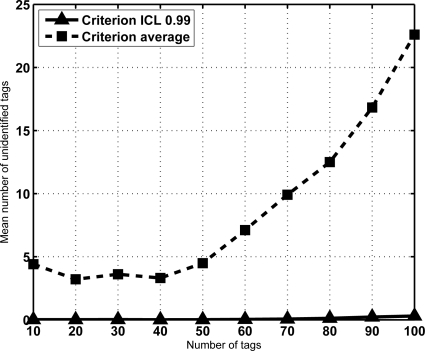
Mean number of unidentified tags for *K* = 16. Setting the average number of slots provided by Equation (5) as sojourn time, curve called “Criterion average” results in a large number of tags lost.

The above-mentioned results indicate that in a mixed scenario the sojourn time of the tags must be much larger than the average time required for reading, specially for large groups of tags. This might result counterintuitive. A justification is provided in Figure 5. It shows the CDF of *V_N_* for a configuration with *N* = 100 and *K* = 16. For a given sojourn time, it depicts the probability that all tags in the group have been identified. In this figure, the average identification time is marked, and its corresponding probability. The result is notable, since it means that when setting this value as the sojourn time more than 50% of the sets would leave with some unidentified tags. This is not acceptable in most RFID facilities. From another point of view, Figure 6 shows a comparative of the average number of lost tags (*L̄*) using both criteria for the sojourn coverage. *L̄* is the expectation of the number of unidentified tags at frame *s*, corresponding to the probability distribution *π*^(*s*)^. Namely,

(8) $$\bar{L}=\sum_{i=0}^{N}\left(N-i\right)\pi_{i}^{\left(s\right)}$$

Clearly, the average number of lost tags grows as the population increases if the sojourn time is the average identification time. Moreover, the average number of lost tags with the criterion of ICL level set to 0.99 is almost negligible. Result trends are similar regardless of the frame length *K*, and for larger tag sets.

Summarizing, there is a trade off between the sojourn time required (conversely, in the speed of processing of the items) and the possibility of losing tags in a RFID installation. Previous results allow to select the desired operational point in this trade off.

## 5. Splitting Strategy

Let us recall from previous section that the differential sojourn time required to achieve a given ICL level increases with the size *N* of the set of tags. This observation suggests that if items could be rearranged in smaller subsets the overall sojourn time (to identify all the subsets) may decrease, achieving the same global ICL confidence. In this section the influence of this effect is studied for mixed scenarios. Specifically, the aim is at deciding if splitting the set of tags reduces the overall identification time, and computing the optimal configuration of the subsets.

In the splitting model, the *N* tags of a group are assumed to be uniformly redistributed into *J* smaller subsets of size $N\prime =\frac{N}{J}$ tags (for simplicity we consider a perfect partition, *i.e.*, *N′* is integer). Let *V′_N_* denote the (random) identification time of the *J* subsets. It can be expressed as,

(9) $$V_{N}^{\prime }=\sum_{i=1}^{J}V_{N\prime }$$

**Figure 7.**
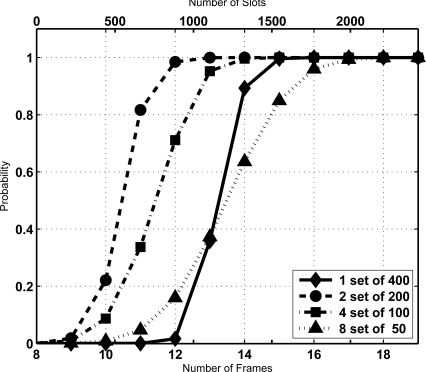
Comparison of the CDF of the identification time for *N* = 400, *J* = (1, 2, 3, 4), and *K* = 128.

Since the reading time of one subset is independent from the reading time of any other subset the mass probability function of *V′_N_* is just,

(10) $$P[V_{N}^{\prime }=s]=P\left[V_{N\prime }=s\right]*\underset{\ldots }{J}*P\left[V_{N\prime }=s\right]$$

where the * operator denotes convolution. From the previous equation the cumulative distribution function of *V′_N_* is obtained. As an example of the results, Figure 7 shows the CDF of the identification time (measured in frames or in slots) achieved when a population of *N* = 400 tags is split into *J* = 2, 4 and 8 subsets (plus the original set of 400 tags), for a frame length of *K* = 128. In this scenario, for the same ICL level as in the previous numerical examples, the configuration of two sets of 200 tags provides the best results, being the first configuration able to surpass the ICL = 0.99 at frame 13. Therefore, these results confirm our previous discussion, that is, the fact that the derivative of the sojourn time increases with *N* effectively indicates that reducing the size of the set increases the reading efficiency. Hence, splitting is useful and the next step is to find the optimal size of subsets.

Besides, due to the partition of the sets a time lapse between each subset should be reckoned on in a complete model of the mixed scenario. Let *τ* denote this time lapse considered between two subsets. Parameter *τ* can be regarded either as a safeguard time for the identification of tags (e.g., a temporal spacing in a conveyor belt) or an operational parameter that takes into account an overhead for splitting the sets (e.g., modelling a robotic arm that removes a subset from the reading area and introduces a new one). With this restriction into account, the overall identification time (*T*) is given in next equation. For simplicity, *τ* is considered as an integer number of frames (conversely, slots).

(11) $$T=V_{N}^{\prime }+\left(J-1\right)\tau$$

And its mass probability function is,

(12) $$P\left[T=s\right]=P\left[V_{N}^{\prime }=s-\left(J-1\right)\tau \right]$$

**Figure 8.**
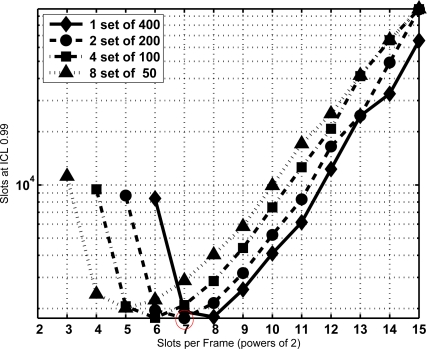
Total identification time in number of slots required with ICL = 0.99, *N* = 400, τ= 0 and its subsets.

**Figure 9.**
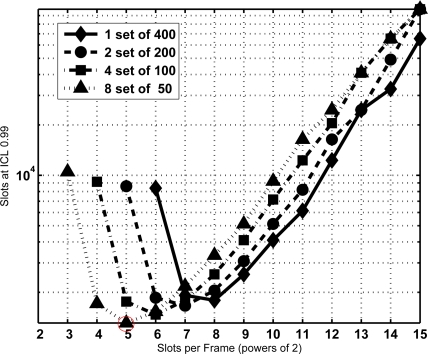
Total identification time in number of slots required with ICL = 0.99, *N* = 400, τ = 100 and its subsets.

**Figure 10.**
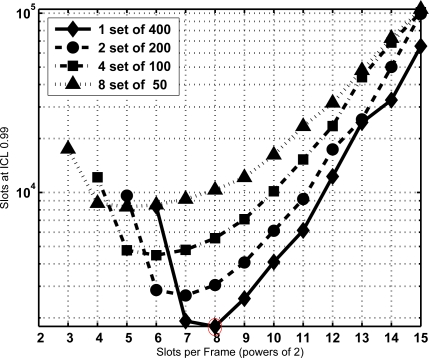
Total identification time in number of slots required with ICL = 0.99, *N* = 400, τ = 1,000 and its subsets.

From the former equation the CDF of *T* is extracted and the minimum sojourn time is directly determined with the same procedure as Section 3.2. The minimum sojourn time has been evaluated for different subset sizes and *τ* settings. Figures 8, 9 and 10 show the results for *N* = 400 tags, *J* = 1, 2, 3 and 4, *τ* = 0, 100, and 1,000 slots, ICL = 0.99, and different frame lengths *K* = 2*^x^*, being *x* ∈ [3, 4. . . , 15]. As can be seen, in both figures there is an optimal configuration for each value of the frame length *K*, and a global optimum for a particular *K*. In particular, Figure 9 shows that this global optimum is achieved with *x* = 5 (*i.e.*, *K* = 32), and 8 subsets of 50 tags. For larger values of the overhead *τ*, as in Figure 10, using a single set becomes a better option.

## 6. Conclusions

This paper provides sound criteria for the configuration of RFID facilities in mixed scenarios. Since in those scenarios it is not possible to ensure a successful identification of all tags in every set, a trade off must be established: the lower the probability of losing some tag in a group, the greater the minimum sojourn time required for the identification, and vice versa. Section 4 was devoted to analyze this trade off. Optimal configurations (in terms of minimal sojourn time) are provided for different configuration setups.

Moreover, the results analyzed in Section 4 strongly suggest the splitting of tag sets in smaller subsets to achieve the same overall tag loss probability, but with reduced sojourn time. Section 5 addresses this issue. A model that takes into account a safeguard time between tag subsets is introduced. Results show that the splitting strategy is preferred given that the inter-arrival time between sets (*τ*) is sufficiently short.

## Figures and Tables

**Table 1.** Number of frames needed to achieve *ICL* = 0.99 for frame lengths *K* = 16, 32, 64 and number of tags *N* = 50, 100, 200, 300.

Frame length (slots)	Number of tags			

	50	100	200	300

16	17	169	∞	∞
32	7	16	144	∞
64	5	7	15	41
